# Extracellular enzyme production and cheating in *Pseudomonas fluorescens* depend on diffusion rates

**DOI:** 10.3389/fmicb.2014.00169

**Published:** 2014-04-11

**Authors:** Steven D. Allison, Lucy Lu, Alyssa G. Kent, Adam C. Martiny

**Affiliations:** ^1^Department of Ecology and Evolutionary Biology, School of Biological Sciences, University of California-IrvineIrvine, CA, USA; ^2^Department of Earth System Science, School of Physical Sciences, University of California-IrvineIrvine, CA, USA

**Keywords:** cheating, diffusion, extracellular enzyme, protease, protein, *Pseudomonas fluorescens*, social evolution, spatial structure

## Abstract

Bacteria produce extracellular enzymes to obtain resources from complex chemical substrates, but this strategy is vulnerable to cheating by cells that take up reaction products without paying the cost of enzyme production. We hypothesized that cheating would suppress enzyme production in co-cultures of cheater and producer bacteria, particularly under well-mixed conditions. To test this hypothesis, we monitored protease expression and frequencies of *Pseudomonas fluorescens* producer and cheater genotypes over time in mixed liquid cultures and on agar plates. In mixed culture inoculated with equal frequencies of cheaters and producers, enzyme concentration declined to zero after 20 days, consistent with our hypothesis. We observed a similar decline in cultures inoculated with producers only, suggesting that cheater mutants arose *de novo* and swept the population. DNA sequencing showed that genetic changes most likely occurred outside the protease operon. In one experimental replicate, the population regained the ability to produce protease, likely due to further genetic changes or population dynamics. Under spatially structured conditions on agar plates, cheaters did not sweep the population. Instead, we observed a significant increase in the variation of enzyme activity levels expressed by clones isolated from the population. Together these results suggest that restricted diffusion favors a diversity of enzyme production strategies. In contrast, well-mixed conditions favor population sweeps by cheater strains, consistent with theoretical predictions. Cheater and producer strategies likely coexist in natural environments with the frequency of cheating increasing with diffusion rate.

## INTRODUCTION

The breakdown of complex organic matter is a fundamental biogeochemical process that is largely controlled by microbes. Living biomass contains a wide range of polymeric macromolecules, including proteins, nucleic acids, and structural carbohydrates (Stevenson, 1994; Kögel-Knabner, 2002; Lee et al., 2004). Microbes produce extracellular enzymes to degrade these compounds into smaller molecules that can be taken up across the cell membrane and metabolized (Burns, 1982; Sinsabaugh, 1994). Thus carbon and nutrient cycles in ecosystems depend on microbial extracellular enzymes.

Microbes can benefit from enzyme production by accessing energy and nutrients in complex compounds, but this strategy also requires a substantial resource investment in enzyme synthesis and excretion (Frankena et al., 1988). Enzyme production may consume ~1–5% of bacterial productivity (Giuseppin et al., 1993; Christiansen and Nielsen, 2002), and also requires large amounts of nitrogen because enzymes have C:N ratios of ~3:1 (Sterner and Elser, 2002).

Extracellular enzymes belong to a class of chemical compounds known as “public goods” because they are costly to individual microbes but increase resource availability for other organisms. Public goods include compounds like signaling molecules, antibiotics, siderophores, and secreted proteins (West et al., 2007). The production of public goods represents an evolutionary conundrum because natural selection should favor “cheater” variants that exploit public goods without paying the cost of production (Velicer, 2003; Travisano and Velicer, 2004). By avoiding this cost, cheaters can gain a competitive advantage and increase their individual fitness and population size relative to enzyme producers.

Theoretical studies suggest that cheaters can suppress enzyme producers and reduce substrate degradation rates in model systems (Allison, 2005). Theory also shows that cheating and cooperation depend on spatial structure (Nowak and May, 1992; Wakano et al., 2009). Under well-mixed conditions, cheaters have equal access to the benefits of enzyme production and readily out-compete enzyme producers. In contrast, restricted diffusion causes the products of the enzymatic reaction to remain spatially close to enzyme producers, giving producers a local competitive advantage against cheaters. Thus limited diffusion and resulting spatial structure promote coexistence of cheater and producer strategies.

Similar patterns have been observed with other public goods. For instance, colicin-producing, colicin-resistant, and colicin-susceptible strains of *E. coli* can coexist in spatially structured but not well-mixed environments (Chao and Levin, 1981; Durrett and Levin, 1997; Kerr et al., 2002). Similarly, multiple strains of *Pseudomonas fluorescens* can evolve and coexist in spatially heterogeneous microcosms (Rainey and Travisano, 1998), and siderophore production by *P. aeruginosa* is less susceptible to cheating when dispersal is limited (Kümmerli et al., 2009a). Christensen et al. (2002) showed that when growing on benzyl alcohol, *P. putida* and *Acinetobacter* coexist in a biofilm but not in a well-mixed environment.

Although some empirical studies are consistent with cheating in extracellular enzyme systems, few studies have confirmed that reductions in enzymatic function are due to cheaters. For example, Worm et al. (2000) found that a protease-negative *P. fluorescens* strain survived on complex protein as a primary N source when grown with a wild-type strain that was protease-positive. However, it was not clear if the putative cheater in this system had a negative impact on enzyme production. Another study by Romaní et al. (2006) found that bacteria had a negative impact on enzyme-producing fungi that were decomposing plant litter in aquatic mesocosms. In this case, negative interactions could have resulted from cheating or some other mechanism, such as toxin production (Mille-Lindblom et al., 2006).

The predicted negative impact of cheaters on enzyme producers has been demonstrated via controlled competition experiments with genetically manipulated microbes. Gore et al. (2009) used yeast strains lacking the invertase gene as an extracellular enzyme cheater and defined a range of conditions under which cheater and producer strains could coexist. Cheaters were demonstrated to have a negative impact on producer growth, but mainly at high population densities. At low population densities, invertase producers were able to maintain a growth advantage through a small (~1%) positive differential in access to hydrolysis products.

The objective of our study was to analyze the effects of diffusion, cheating, and evolution in extracellular enzyme systems. Although spatial structure is known to promote diversity in many microbial systems, theoretical predictions about the effect of diffusion on enzyme cheating have not been confirmed. Based on findings with similar public goods (Kümmerli et al., 2009b), we hypothesized that restricted diffusion should allow enzyme producers to preferentially access reaction products and thereby coexist with cheaters. In contrast, we expected cheaters to outcompete enzyme producers under well-mixed conditions in liquid cultures. We tested these hypotheses using a protease-producing strain of *P. fluorescens* and an isogenic knockout strain that was protease-negative (the cheater). We tracked cheater-producer dynamics over time in liquid culture or on agar plates with transfers to new medium every few days. This design, commonly used in experimental evolution studies (Elena and Lenski, 2003), also allowed us to observe the *de novo* emergence of new enzyme production phenotypes.

## MATERIALS AND METHODS

### STRAINS

We obtained strains of *P. fluorescens* ON2 from Dr. Ole Nybroe’s laboratory at the University of Copenhagen, Denmark. The original strain ON2 was isolated from a freshwater sediment and subjected to genetic manipulation via electroporation with plasmid pJBA28. This plasmid contains a pUTmini-Tn5-*gfp* gene cassette with a kanamycin (kan) resistance marker (Christoffersen et al., 1997; Worm et al., 2000). The wild-type “producer” strain ON2 secretes a single extracellular metalloprotease, whereas strain ON2-pd5 (the “cheater”) contains the Tn5 gene cassette and does not secrete protease. Protease phenotype was screened by selective plating on kan agar containing skim milk; the cheater strain was unable to metabolize skim milk. Subsequent sequencing of the cheater strain showed that the transposon was inserted downstream of the protease structural gene and likely interfered with expression of an ABC transporter required for protease secretion (Worm et al., 2000).

### MEDIA

Strains were grown on either M63 medium or casein medium. The base M63 medium (pH 7.0) contained 2.0 g/l (NH_4_)_2_SO_4_, 13.6 g/l KH_2_PO_4_, 0.5 mg/l FeSO_4_[H_2_O]_7_, and 0.2% (w/v) glucose. Casein medium contained 0.4% (w/v) casein in diluted M63 medium (0.25 × in deionized water). Base and casein media were supplemented with 1 mM MgSO_4_, 0.35 mM CaCl_2_, and 50 μg/ml ampicillin. Solid media for plate experiments contained 1.5% agar.

### EXPERIMENTAL DESIGN

For experiments in shaking flasks, cells were grown overnight in M63 medium to an OD_600_ of ~1 and inoculated into triplicate 50 ml flasks containing 10 ml casein medium. One set of triplicates was inoculated with overnight culture containing only cheater cells. We added 5 mg/l proteinase K to these flasks to hydrolyze casein and allow for cheater growth. This concentration of proteinase was equivalent to the protease present in producer cultures at stationary phase. These flasks served as a control for cheater growth and allowed us to confirm that the Tn5 insertion was maintained in the absence of kan for the duration of the experiment. Another set of triplicate flasks contained casein medium and was inoculated with a mixture of 50% cheater cells and 50% producer cells to examine competition between the strains. A final set of triplicate flasks also contained casein medium but was inoculated with only producer cells. This treatment was designed to test whether protease function was maintained in the absence of inoculated cheaters. For all three experiments, starting OD_600_ in each of the triplicate flasks was 0.01.

All flasks were maintained at 28°C in a dark incubator with orbital shaking at 200 rpm. After the cultures reached stationary phase, 100 μl culture fluid was transferred to a new flask containing 10 ml fresh medium. For trial 1 of this experiment, transfers were done every 1 or 2 days for 23 days depending on the availability of laboratory staff. For the second trial, transfers were standardized to occur every 48 hours for 30 days. Optical densities at stationary phase were ~1.0, which corresponds to ~3 × 10^9^ cells/ml (Worm et al., 2000). Given that the culture was diluted 100-fold at each transfer, growth to stationary phase represents approximately 6.64 bacterial generations. Relative abundances of ON2-pd5 were determined at several time points by diluting the culture fluid, plating out on LB agar plates (all with 50 μg/ml ampicillin), and counting the number of colonies on plates with and without kan (25 μg/ml).

For the agar plate experiments, triplicate plates were inoculated with ~6.6 × 10^6^ producer cells or a 50–50 mixture of cheaters and producers, as in the flask experiment. Because growth was slower on agar, transfers occurred every 3 days for 81 days. Cells were transferred to new agar medium by contact plating. Our shaking flask experiment showed that after a period of time, protease phenotype did not necessarily correspond to kan resistance phenotype. Therefore, we assayed protease expression directly on clones isolated from agar plates to determine the frequencies of cheaters and producers in this experiment.

### PROTEASE ASSAYS

For the shaking flask experiment, we used the colorimetric azocasein method (Tomarelli et al., 1949) to assay protease activities in the culture fluid at 2–7 day intervals during each trial. Protease activities were expressed as enzyme concentrations (proteinase K equivalents) based on a standard curve of absorbance versus proteinase K concentration (440 nm after 60 min incubation at 37°C). We also measured the frequency of cheater and producer phenotypes on days 12 and 24 of the second trial in flasks inoculated with producers only. The culture fluid was diluted and plated out on agar, and 22 individual colonies were picked and assayed for protease activity with the azocasein method after 48 h growth on casein medium. For the agar plate experiment, we used a high-throughput fluorimetric assay (Pierce^®^ Fluorescent Protease Assay, Thermo Scientific) to assay protease expression in 100s of randomly isolated clones.

### GENETIC ANALYSES

To identify possible genetic variation underlying changes in protease phenotype, we extracted and sequenced genomic DNA from all replicates of trial 2 (day 30) in the shaking flask experiment. We also extracted and sequenced the genomic DNA of the ancestral strain *P. fluorescens* ON2 for a total of 10 libraries. Genomic DNA was extracted with the Wizard Genomic DNA Purification Kit (Promega) according to the manufacturer’s instructions, sheared to ~300 bp with the Covaris S2 System, and barcoded with the TruSeq DNA sample prep kit (Illumina). Fragments were sequenced on the Illumina platform at the UC Irvine Genomics High-Throughput Facility. The resulting reads from each library were assembled into contigs using PRICE (Ruby et al., 2013), Velvet (Zerbino and Birney, 2008), and Minimus (Sommer et al., 2007). A 94 kbp contig (GenBank accession KJ540107) from the ON2 assembly was used as a reference sequence for mapping Illumina reads. This contig contained the extracellular protease operon as confirmed by BLASTn alignment to a 434 bp sequence upstream of the Tn5 insertion point provided by Dr. Nybroe (GenBank accession KJ540106). Prior to mapping, all 100 bp reads from ON2 and each of the shaking flask libraries were trimmed to 40 bp with the FAST-X Toolkit (http://hannonlab.cshl.edu/fastx_toolkit/) and aligned to the reference contig using BLASTn with a >90% sequence identity cutoff and hit length >30 bp. BLAST matches were mapped to the reference contig using Geneious 7.0.6 (Biomatters Ltd., http://www.geneious.com). Geneious software was used to search for genetic differences between the reference contig and the mapped sequences from each experimental library. Mean coverage after mapping ranged from 120 to 190× within libraries with an overall average of ~160×.

### STATISTICAL ANALYSES

Optical densities were compared across inoculation treatments using analysis of covariance with time as the covariate. We used Tukey tests to determine if changes in optical densities over time were significantly different across treatments. For the agar plate experiment, we calculated the frequencies of cheaters versus producers based on relative enzyme expression of isolated clones. Background fluorescence of the substrate and medium was subtracted from the fluorescence of each clone to calculate net fluorescence. Relative expression values were calculated by dividing the net fluorescence of each clone by the maximum net fluorescence observed at a given transfer. For the agar plate experiment, clones with relative expression values >0.56 were considered producers whereas clones below this value were classified as cheaters. We also calculated the coefficient of variation in relative protease expression across all clones at each time point and used linear regression to determine if there was a significant increase in variation over time.

## RESULTS

### SHAKING FLASKS

**FIGURE 1:**
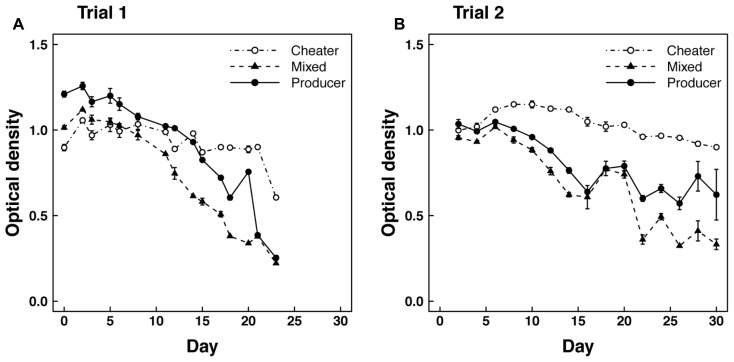
**Optical density over time in (A) Trial 1 and (B) Trial 2 for cheaters grown alone on hydrolyzed casein and producers grown alone or mixed (50–50%) with cheaters on casein medium**.

In trial 1 with shaking flasks, we observed significant (*P* < 0.001) declines in optical density from >1.0 to <0.3 over a 23 day period in mixed and producer cultures growing on casein medium (**Figure 1A**). We also observed a slight but significant decline in the optical density of cheater cultures growing on hydrolyzed casein (*P* < 0.01). However, the declines in the mixed and producer cultures were significantly greater (*P* < 0.001) than in the cheater cultures, which had optical densities near 0.9 for most of the trial. Producer optical densities were higher than mixed cultures until day 21 when they converged on similar values, but the rate of decline was not significantly different between the two treatments.

**FIGURE 2:**
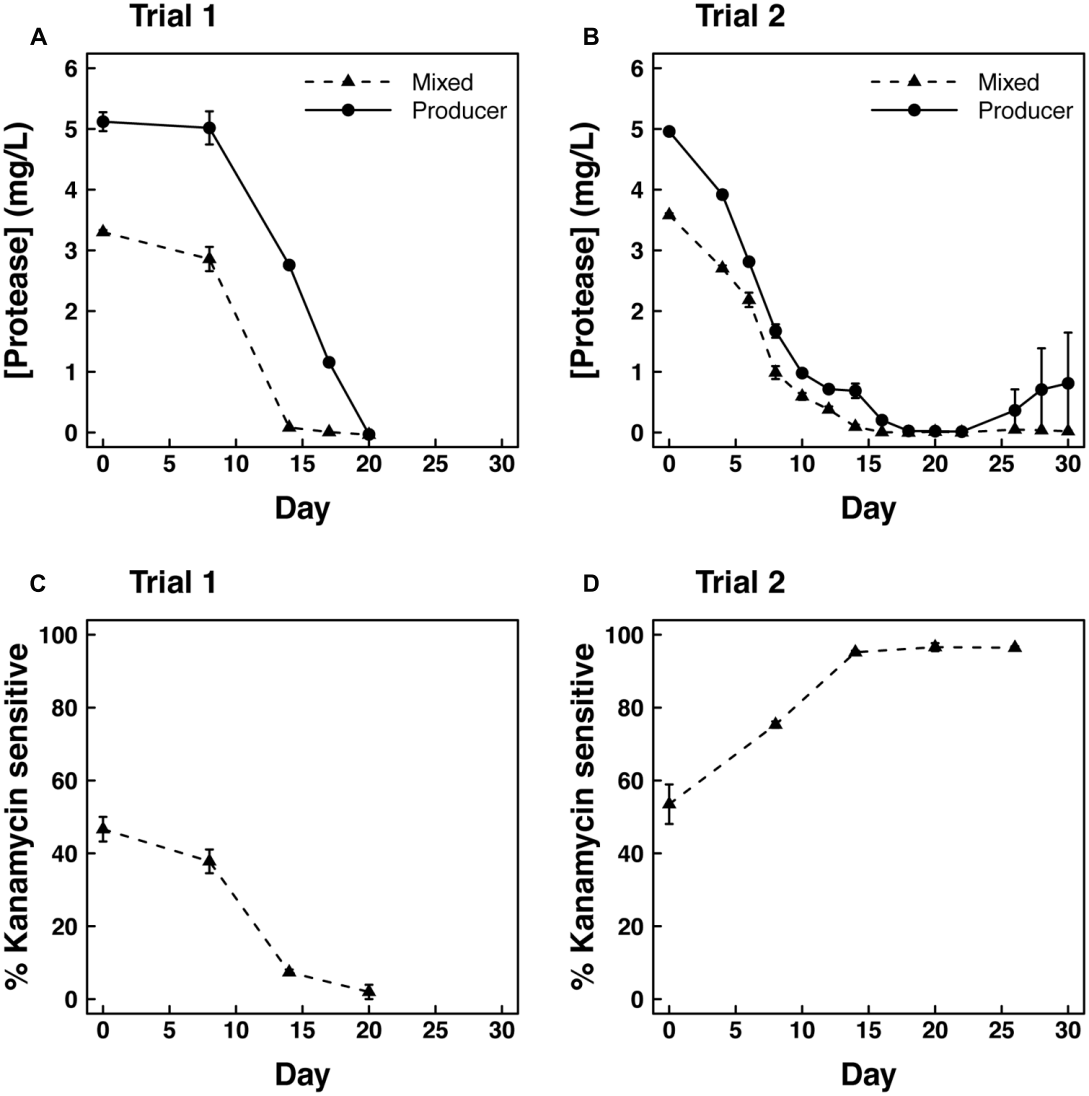
**Protease concentration (mg/l proteinase K equivalents) over time in (A) Trial 1 and (B) Trial 2 for producers grown alone or mixed (50–50%) with cheaters.** Kanamycin sensitive fraction of isolated clones over time in **(C)** Trial 1 and **(D)** Trial 2 in the mixed culture.

Concurrent with the decline in optical density, protease concentration and kan sensitivity also declined during trial 1 in cultures growing on casein medium. Protease was initially higher in producer cultures than in mixed cultures, but neither culture showed detectable protease by day 20 of the trial (**Figure 2A**). The fraction of kan sensitive clones indicative of the producer genotype also declined from 50% to nearly 0% during this time period (**Figure 2C**).

The second mixed flask trial was similar to the first in overall patterns of optical density (**Figure 1B**) and protease concentration (**Figure 2B**). However, the rate of decline in optical density of the producer culture was not as large as in the mixed culture (*P* < 0.05). This difference is largely due to the recovery of protease concentration in one of the three replicate producer flasks. In this flask, optical density and protease concentration recovered to levels of 0.9 and 14.4, respectively, leading to increased variability in these parameters after 28 days (**Figures 1B** and **2B**). Also in contrast to the first trial, kan sensitivity indicative of the producer genotype increased to nearly 100% after 28 days in the mixed cultures (**Figure 2D**). However, assays on individual clones isolated from producer cultures revealed that ~76 and ~86% of the cells were negative for protease expression on days 12 and 24 of trial 2, respectively.

### GENETIC ANALYSES

We did not find evidence for genetic changes in the protease operon or surrounding regions that could account for observed differences in protease phenotype. Although we detected multiple genetic variants with frequencies >10% in each of the mapped genomic libraries from trial 2, these variants were also present in the ancestral ON2 strain. Our genetic analyses were clearly capable of detecting genetic variation because sequences containing Tn5 genetic material were found in all libraries that contained the ON2-pd5 cheater strain.

### AGAR PLATES

**FIGURE 3:**
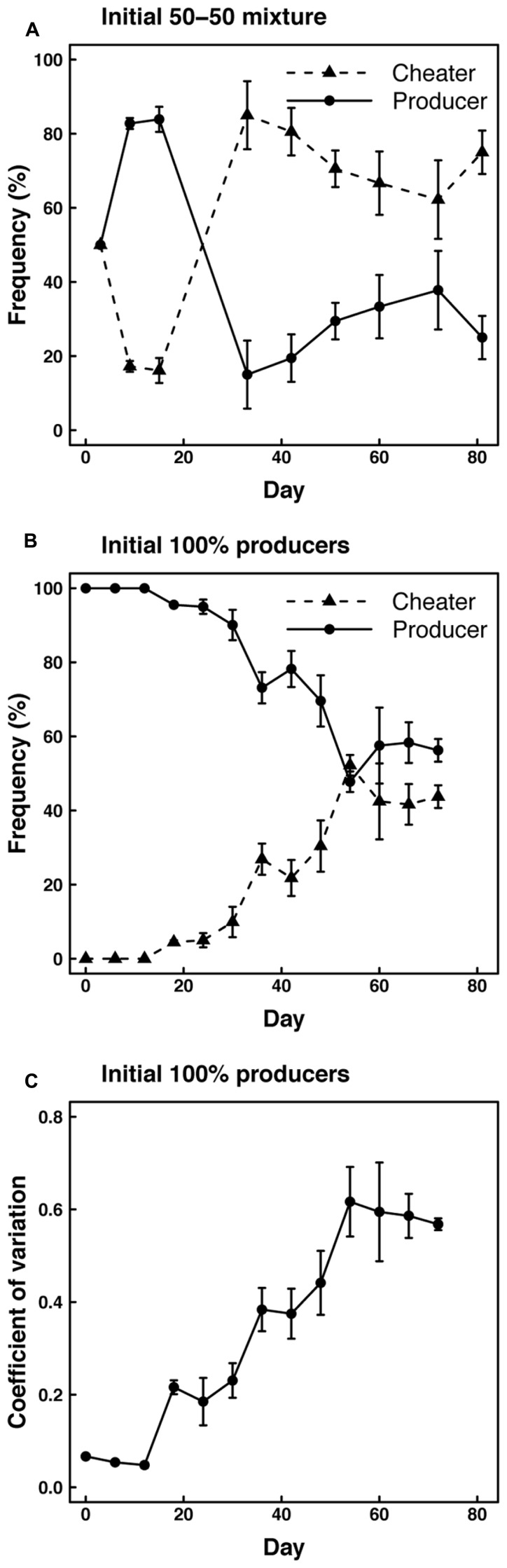
**Frequencies of cheaters and producers based on protease expression in experiments with (A) cheaters and producers (50–50%) or only producers (B) initially inoculated onto agar plates. (C)** Coefficient of variation in protease expression level based on clones isolated from the agar plates inoculated with producers only.

To assess the effect of spatial structure and restricted diffusion on enzyme production, we monitored the frequency of cheater and producer phenotypes in cultures maintained on agar plates. When starting with 50% cheater and 50% producer genotypes, cultures on plates were initially dominated by producer phenotypes (>80%), but cheater phenotypes became dominant after 30 days and remained dominant through the end of the experiment (**Figure 3A**). Similarly, cultures started with 100% producers were dominated by producer phenotypes through the first few transfers, but cheater phenotypes became more abundant over time (**Figure 3B**). After 50 days, roughly equal frequencies of cheater and producer phenotypes were maintained on agar plates. Consistent with these results, we observed a significant (*P* < 0.001) increase over time in the coefficient of variation for protease production among clones isolated from the agar plates initiated with producers only (**Figure 3C**). These results indicate an increasing diversity of enzyme expression levels over the course of the experiment.

## DISCUSSION

Data from both trials under shaking conditions support the hypothesis that cheater phenotypes have a selective advantage. In the first trial, a loss of protease function over 23 days was accompanied by a loss of the kan sensitive genotype characteristic of our producer strain. Under shaking conditions, protease-negative mutants likely have a selective advantage because they do not pay the costs of enzyme production, yet they can access casein hydrolysis products (Allison, 2005).

Even if no cheater knockouts were inoculated into the initial culture, we still observed loss of protease function over time (**Figures 2A,B**). This pattern could have arisen from *de novo* genetic mutations that conferred a protease-negative phenotype. Our genetic analyses suggest that any such mutations must have occurred outside the protease operon. We did not observe genetic variation in or near the operon that could account for loss of protease function. Still, this result does not rule out a genetic basis for the change in phenotype. Mutations in many enzyme and regulatory genes can have pleiotropic effects that increase fitness in bacteria (Hottes et al., 2013). One or more of these distant mutations could have reduced protease expression in our study.

Our second trial under shaking conditions suggests that cheaters may arise by multiple evolutionary pathways with different fitness consequences. In this trial, we observed a decline in protease expression in the 50–50 mixed culture, but there was no associated loss of kan sensitivity. This pattern suggests that *de novo* mutations in the wild-type strain may have generated cheater strains with higher fitness than our original knockout strain. Since our knockout was generated by random transposon insertion, it is not surprising that other genetic changes affecting protease production would result in higher fitness. The transposon in our knockout was inserted downstream of the protease gene but upstream of genes involved with protease secretion, meaning that our ON2-pd5 cheater strain may still incur the costs of transcription and translation for other genes in the protease operon. *De novo* mutations that reduce or eliminate these costs would be expected to confer higher fitness relative to our original cheater knockout.

It is somewhat surprising that we observed distinct evolutionary outcomes in trial 1 versus trial 2. Cheaters derived from the wild-type swept all three replicate flasks in the 50–50 mixture of Trial 2, but the original cheater knockout (or its descendants) swept the populations in Trial 1. With only three replicates, we cannot rule out that the different outcomes were due to chance (*P* = 0.1; Fischer’s exact test). Alternatively, it is possible that the selective environment differed between the two trials. In particular, the transfers were not regularly spaced for the first trial (every 1–2 days) as they were for the second trial (every 2 days). On average, the cells in trial 1 spent a larger fraction of time growing on fresh medium and less time in stationary phase, which would alter the chemical environment and selective pressures in the flasks.

The results from the liquid producer culture in trial 2 suggest that the loss of protease function is reversible. Protease loss during the first 25 days of the trial reduced the availability of casein hydrolysis products. Under these conditions, protease-producing variants are favored because proteolysis increases resource availability for growth while cheater densities are relatively low. The protease-positive phenotype that emerged after 25 days could have resulted from compensatory mutations or positive selection on rare producer genotypes that survived the population bottleneck on day 22.

Alternatively, loss and recovery of protease function could have resulted from changes in gene expression. However, this mechanism is unlikely given that most clones isolated on days 12 and 24 from producer flasks in trial 2 did not express protease even after plating on agar and regrowth on casein medium. Furthermore, the complete loss of protease function and crash of producer populations probably would not have occurred if gene expression were flexible.

The recovery of protease expression is indicative of frequency-dependent selection, whereby producers are favored at low population densities and cheaters are favored at high densities (Greig and Travisano, 2004). Theoretical studies show that frequency-dependent selection may be driven by spatial structure that allows rare producers to gain preferential access to reaction products (Durrett and Levin, 1994; Wakano et al., 2009). Spatial structure is important because rare producers under perfectly mixed conditions should be out-competed by cheaters since both variants would have equal access to (dilute) reaction products. There may have been some undetected spatial structure in our flasks that facilitated frequency-dependent selection, such as biofilm formation. This spatial structure was probably limited since we only observed a recovery of protease expression in one flask of the six included in our trials. Clearly defining the role of spatial structure and frequency-dependent selection in shaking flasks would require a larger number of replicates and experiments lasting more than 30 days.

Results from maintaining cultures on agar plates support the hypothesis that limitations on diffusion enhance the coexistence of cheater and producer strains. Frequency variations from the 50–50 mixture are suggestive of oscillations predicted by theoretical models under intermediate diffusion levels (Allison, 2005). Although additional experiments would be required to confirm the consistency and duration of these cycles, they are consistent with the theoretical effect of spatial structure on frequency-dependent selection (Durrett and Levin, 1994).

As in the shaken flask experiment, the agar plate experiment showed evidence for evolution of phenotypes with reduced protease expression. However, the shaking flasks were subject to sweeps by cheaters with undetectable levels of protease expression, whereas the agar plates hosted strains with a continuum of enzyme production levels that we arbitrarily classified into cheaters versus producers. This diversity is evident from the significant increase in the coefficient of variation in protease expression across randomly selected isolates (**Figure 3C**).

Reduced diffusion and increased spatial structure likely both contributed to increased diversity on agar plates. Limits on diffusion allow cheaters and cooperators to coexist because cooperators gain preferential access to the benefits of public goods production (Kümmerli et al., 2009b). In addition, the formation of colonies on agar generates spatial gradients in enzyme, substrate, product, and by-product concentrations. This spatial structure likely leads to differentiation in enzyme production strategies by increasing the number of chemical resource niches. Such a relationship between growth, spatial structure, and strain diversity has been observed with *P. fluorescens* growing in unshaken flasks (Rainey and Travisano, 1998).

Our results have implications for the maintenance of microbial diversity and ecological function in environmental settings. Few natural environments are completely well-mixed, and therefore the selective sweeps that may have occurred in our shaking flasks are probably uncommon. Even in aquatic and marine ecosystems, particulate organic matter generates spatial heterogeneity that could select for a range of enzyme production strategies (Smith et al., 1992). Biofilm formation is common on particles and surfaces in aqueous environments, further increasing chemical and spatial heterogeneity that can facilitate coexistence (Hansen et al., 2007; Cordero et al., 2012). Multi-phase environments such as soils and sediments are likely to have even lower diffusion rates and greater spatial heterogeneity than our agar plate system (Moldrup et al., 2001). Therefore it is likely that evolutionary processes and spatially mediated coexistence are constantly maintaining microbial diversity in these environments.

Although we identified important mechanisms for maintaining diversity and function despite the potential for cheating, our results also suggest that cheaters play a role in many microbial systems. At least in aquatic and saturated systems most similar to our experimental conditions, cheaters are probably always present to some degree. Broadly defined, cheating need not involve a complete loss of enzyme expression or other function. A reduction in the amount of enzyme production relative to neighboring cells is also a form of cheating if the enzyme benefits are distributed equally but the costs are unequal.

Regardless of its form and magnitude, cheating can impact ecological processes, such as hydrolysis rates for biomolecules. In the case of enzyme production strategies, greater diversity and overall microbial growth may not translate into maximal rates of hydrolysis (Folse and Allison, 2012). Gore et al. (2009) showed that adding glucose (a reaction product of invertase) actually reduces the equilibrium fraction of invertase producers in the population and therefore the hydrolysis rate of sucrose, the invertase substrate. Although our experiments only examined proteolysis under controlled conditions, our results raise the possibility that enzyme-driven decomposition may not increase with diversity as has been observed with other ecosystem functions (Tilman et al., 2006). The evolutionary and ecological processes that generate diversity need not maximize biogeochemical rates because of the constant selective pressure for cheating.

## AUTHOR CONTRIBUTIONS

Steven D. Allison conceived the project, designed experiments, analyzed data, and wrote the manuscript. Lucy Lu conducted the experiments, analyzed data, and helped write the manuscript. Alyssa G. Kent and Adam C. Martiny conducted the genetic analyses and helped write the manuscript.

## Conflict of Interest Statement

The authors declare that the research was conducted in the absence of any commercial or financial relationships that could be construed as a potential conflict of interest.
